# The Early Evidences of Future Malocclusion, and the Advantages of Immediate Treatment

**Published:** 1914-04-15

**Authors:** Milton Tate Watson


### THE EARLY EVIDENCES OF FUTURE MALOCCLUSION, AND THE ADVANTAGES OF IMMEDIATE TREATMENT.

BY MILTON TATE WATSON, D.D.S.

In as few words as possible, I shall endeavor to show the modern trend in orthodontia. Like all other branches of dentistry, orthodontia is gradually coming under a more rational influence, and the difficulty of successfully correcting marked malocclusion of several years standing, as well as the difficulty of overcoming the unfortunate effects of these years of maldevelopment, has stimulated all thinking orthodontists, to make earlier diagnoses, that treament may be begun early enough to overcome, at least the major part of the deformity, and prevent its evil influences.

In all cases where an early diagnosis is made, and treatment is begun at four, five or six years of age, we not only lessen to a very marked degree the amount of physical force required to produce the necessary changes, which is an item of considerable importance, but we prevent to a very large degree the nervous stress to which any child is subjected, whose developing teeth are all impacted, as is really the case where the jaws fail to develop sufficiently to provide for a practically normal eruption. In addition to these two important items, the mechanical stimulation of jaw development, in the cases where nature is unable to bring about such development unaided, largely and, in many cases, wholly prevents the unfortunate influence which a restricted oral capacity has on normal nasal respiration. This by the way, in my judgment, is probably the most important individual factor we have to consider.

To Doctor Cryer, of Philadelphia, whose intimate knowledge of the anatomy of the internal face is probably greater than that of any other man in dentistry, is due the credit of clearing up for us the actual reason why widening and lengthening the jaws bring about such a marked improvement in the normal breathing capacity. He has shown by dissections of the frozen cadaver that when the teeth are closed in a subject whose jaws are normally developed, the tongue fills the entire oral cavity, except for a slight space in the very crown of the upper part of the vault of the arch, and yet it does not push back into the throat far enough to displace the soft palate. By dissections made in the same way, with subjects whose dental arches are restricted, he has shown that because of the lack of proper tongue space, when the jaws are closed, the tongue is forced back into the throat and it in turn pushes the soft palate up and back against the post-pharyngeal wall, thus preventing the subject from breathing through the nose, no matter how much nasal space there may be.

You will readily understand from this that the very early enlargement of the dental arches, where such an operation is indicated, prevents a whole cycle of evil influences, which would be effective if this lack of development were allowed to continue for any considerable period of time. The principal advantages gained by early treatment in the order of their importance might be named as follows:

1st. A marked aid in developing the normal breathing capacity, including the far reaching influence this has upon the general health of the child.

2nd. Sufficient space can usually be provided for the eruption of the permanent teeth, though providing this space will not, of course, prevent teeth from erupting in a rotated position if they have started to calcify in this way. It has usually been my experience, however, that if the operation were begun by five years of age that very few, if any, of the teeth will erupt very much rotated.

3rd. At this early age children do not object in the least to being "decorated" with regulating appliances, which is an important consideration later on; as a matter of fact this one thing has often been the cause of failure, as girls, especially, have deliberately removed their retaining appliances, in order to make a better appearance at some social function, and then have left them off so long before returning, that irreparable injury has resulted. This is such a "bug bear" to me that I very rarely take fourteen or fifteen year old patients any more.

4th. The amount of physical force required to stimulate development of the jaws in little four, five or six year old patients is so slight that it is actually a source of almost no discomfort at all, except just while the child is becoming accustomed to the appliances when they are first adjusted. It is literally true that with these little patients we simply stimulate and control the direction of growth, while with older patients, especially those past twelve years of age, the force required is violent—by comparison only, of course.

5th. There can be no question also of the advantage of doing the major part of an orthodontic operation with appliances attached to the temporary teeth, for it is idle to deny the fact that teeth are sometimes injured because of the wearing of regulating appliances. This injury is however, in nearly all cases, due wholly to the lack of proper home care of the teeth, for, of course, if food stuffs are allowed to accumulate about the appliances and to become fermented, the result is an acid condition which will decalcity, superficially at least, the tooth surface with which it is allowed to come in contact. This, naturally, should always be guarded against by properly instructing the patient or nurse regarding the care of the teeth. If in spite of this care injury should result, it is of comparatively little importance as it involves only the temporary teeth.

Now how shall we know at four or five years of age that a child's jaws are not going to develop properly To answer this question from a purely personal experience, I can say to you truthfully that it was not until after nearly ten years of careful observation of developing children, that I reached a positive and definite conclusion.

The simplest cases, from a diagnostic standpoint, at the early age of four or five years, are of course those in which the lower jaw is in either mesial or distal relation to the upper. A condition of this sort really involves such a conspicuous facial deformity, that even the parents are aware of its existence, and need only to be told that such a condition nearly always grows worse instead of better.

A type of case which is much more common, and which all parents, and, as yet, the majority of dentists, look upon as perfectly normal, is that in which the jaw has developed sufficiently to just provide the necessary space for the temporary teeth, but never develops any more. This is the type of case where, because of the lack of space, the lower permanent incisors are often a little delayed in their eruption, and the fond mother is secretly proud of the appearance of her child, as compared with some other child of about the same age, whose temporary teeth have become widely separated, and who has erupted some "great, big permanent front teeth," in good position but, as everybody can see, out of all proportion to the childish face. This same fond mother is, however, rudely awakened a little later on when her child, with his pretty little baby mouth, begins to erupt his permanent incisors very much rotated and completely in lingual relation to the temporary ones, which are often still firmly attached. In the fewest possible words here is the situation. *If the child's jaws were really developing normally, at about four years of age, spaces should begin to appear between all the front teeth above and below; at about five years of age, these spaces should be well marked, and if they are not, then you may feel very certain that the permanent teeth will be irregular.*

Another confusing type is one where an apparently satisfactory width is developing, but where the mesio-distal relation of the dental arches is slightly abnormal, possibly an end to end occlusion of the temporary molars. This type of case will progress to the entire satisfaction of the parents until the permanent cuspids begin to erupt, and then, of course, there is little or no room for them, or if they erupt in advance of the bicuspids then at least one of these teeth will be forced to erupt in a lingual or buccal position. These rather simple and easily understood facts, if you choose to call them such, can be accepted as very safe guides in determining whether or not these little children require attention.

While it is irrelevant so far as the subject of this discussion is concerned, it nevertheless seems an opportune moment to call your attention as general practitioners to a new responsibility which you must not ignore. You may think it should belong to the orthodontist, but circumstances make it difficult for him to see these patients soon enough or to keep them under observation long enough. I refer especially to those cases in which either dental arch, but particularly the lower, shows a marked lack of development in its length, and where this tendency is still in evidence after the orthodontist has developed sufficient space for all of the teeth except the third molars. It sometimes happens that the eruption of these teeth does not stimulate sufficient growth, in the length of the jaw to provide space for them, and in such a case a crowding and overlapping of the anterior teeth will be in evidence as soon as these third molars begin to erupt. If the corrected occlusion has remained so up to the time for the appearance of these teeth and then the anterior teeth begin to show an undesirable change, you should at once make a careful examination to determine whether or not there is space for the third molars, and if there is not, their extraction from that particular jaw should not only be recommended but insisted upon, for otherwise the patient will develop a more or less marked malocclusion, though often of an entirely different type from the original deformity

